# Subclinical impairment of the left atrium is associated with MRI-based lung volume but not with parameters from pulmonary function testing

**DOI:** 10.1038/s41598-024-70777-w

**Published:** 2024-09-10

**Authors:** Charlotte Wintergerst, Roberto Lorbeer, Blerim Mujaj, Bernard E. Bulwer, Susanne Rospleszcz, Esther Askani, Holger Schulz, Stefan Karrasch, Annette Peters, Christopher L. Schlett, Fabian Bamberg, Ricarda von Krüchten

**Affiliations:** 1 Department of Diagnostic and Interventional Radiology, Medical Center‐University of Freiburg, Faculty of Medicine, University of Freiburg, Freiburg, Germany; 2 Department of Radiology, University Hospital, Ludwig-Maximilans-University Hospital Munich, Munich, Germany; 3 German Centre for Cardiovascular Research (DZHK) Partner Site Munich Heart Alliance, Munich, Germany; 4 General Practice, Huisartsenpraktijk, Aalst, Belgium; 5 Cardiovascular Imaging Core Laboratory, Cardiovascular Division, Department of Medicine, Brigham and Women’s Hospital, Boston, MA USA; 6 Institute of Epidemiology, German Research Center for Environmental Health, Helmholtz Zentrum Munich, Neuherberg, Germany; 7 Comprehensive Pneumology Center Munich (CPC-M), Member of the German Center for Lung Research, Munich, Germany; 8 Institute and Outpatient Clinic for Occupational, Social and Environmental Medicine, Ludwig-Maximilians-University Munich, Munich, Germany; 9 Department of Epidemiology, Institute for Medical Information Processing, Biometry, and Epidemiology, Ludwig-Maximilians-University Munich, Munich, Germany; 10 German Center for Diabetes Research (DZD), Partner Site Neuherberg, Neuherberg, Germany; 11 German Center for Cardiovascular Disease Research (DZHK E.V.), Munich, Germany

**Keywords:** Population-based whole-body MRI, Subclinical cardiopulmonary impairment, Heart failure with preserved ejection fraction, Cardiovascular biology, Magnetic resonance imaging

## Abstract

Left atrial (LA) physiology and hemodynamics are intimately connected to cardiac and lung function in health and disease. This study examined the relationship between MRI-based left atrial (LA) size and function with MRI-based lung volume and pulmonary function testing (PFT) parameters in the population-based KORA study cohort of 400 participants without overt cardiovascular disease. MRI quantification assessed LA size/function in sequences with and without ECG synchronization, alongside lung volume. Regression analysis explored the relationship of LA with MRI lung volume and PFT parameters. Among 378 participants (average age 56.3 ± 9.2 years; 42.3% women), non-gated LA size averaged 16.8 cm^2^, while maximal and minimal LA size from gated measurements were 19.6 cm^2^ and 11.9 cm^2^ respectively. The average MRI-derived lung volume was 4.0 L, with PFT showing a total lung capacity of 6.2 L, residual lung volume of 2.1 L, and forced vital capacity of 4.1 L. Multivariate regression analysis, adjusted for age, gender, and cardiovascular risk factors, revealed an inverse association between maximum LA size, non-gated LA, and LA area fraction with lung volume (ß = − 0.03, p = 0.006; ß = − 0.03, p = 0.021; ß = − 0.01, p = 0.012), with no significant association with PFT parameters. This suggests that MRI-based assessment may offer greater sensitivity in detecting subclinical LA impairment than PFT.

## Introduction

Heart failure with preserved ejection fraction (HFpEF) is a significantly different clinical entity from heart failure with reduced ejection fraction (HFrEF)^1^. The pathophysiology of HFpEF is complex, with right ventricular (RV) dysfunction, diastolic dysfunction, and elevated left ventricular (LV) filling pressures leading to elevated left atrium (LA) pressures, LA remodeling, and elevated pulmonary pressures^2,3^. The European Society of Cardiology guidelines list a left atrial volume index > 34 mL/m^2^ as a major diagnostic feature of HFpEF^3^.

Dyspnea in patients with HFpEF is a major symptom resulting from pulmonary congestion and dysfunction due to elevated LV filling pressures^4^. Previous studies have shown that patients with HFpEF have impaired pulmonary function, as assessed through pulmonary function tests such as spirometry, suggesting a common axis of cardiac and pulmonary dysfunction^5,6^. Overlapping pulmonary and cardiac features pose significant challenges for the diagnosis of HFpEF, which often results in delayed diagnosis and treatment^2^. The mortality rate in individuals affected by HFpEF ranges between 10 and 30%, with cardiovascular deaths comprising the primary cause^7^.

Whole-body magnetic resonance imaging (MRI) studies are increasingly being performed for different clinical indications^8^. Furthermore, research-driven population-based whole-body MRI studies are increasingly being performed, allowing the detection of subclinical multiorgan alterations without the use of ionizing radiation^9–11^.

A previous study of the KORA cohort (Cooperative Health Research in the Region of Augsburg) showed an association of MRI-based lung volumes with residual volume and FEV1/FVC (Forced expiratory volume in 1 s/Forced vital capacity, Tiffeneau index) derived from pulmonary function testing (PFT) and also that MRI-based lung volumes were higher in smokers but showed no correlation to traditional cardiovascular risk factors such as hypertension^12^. Another study identified an association between subclinical left and right ventricular impairment and lung volumes assessed through PFT while demonstrating an inverse association with lung volumes derived from MRI-based algorithmic measurements^13^. In the same study cohort, the LA size and function from MRI sequences with and without Electrocardiography (ECG)-gating have been analyzed and an association between cardiovascular risk factors and LA size and function from MRI sequences with and without ECG-gating was confirmed^14^. To date, no published study has been reported to analyze the relationship between LA size and function, and lung function parameters assessed by MRI and PFT. The driving hypothesis of this study is that, even in cardiovascularly healthy subjects, subclinical alterations of the left atrial and pulmonary axis may be present and detectable through PFT or MRI. The aim of this study was thus to investigate the associations between LA size and function, and lung volume derived from MRI. Additionally, we aimed to examine the associations between LA parameters, and lung function parameters assessed through PFT in a population free of overt cardiovascular disease.

## Materials and methods

### Study population

**Fig. 1:**
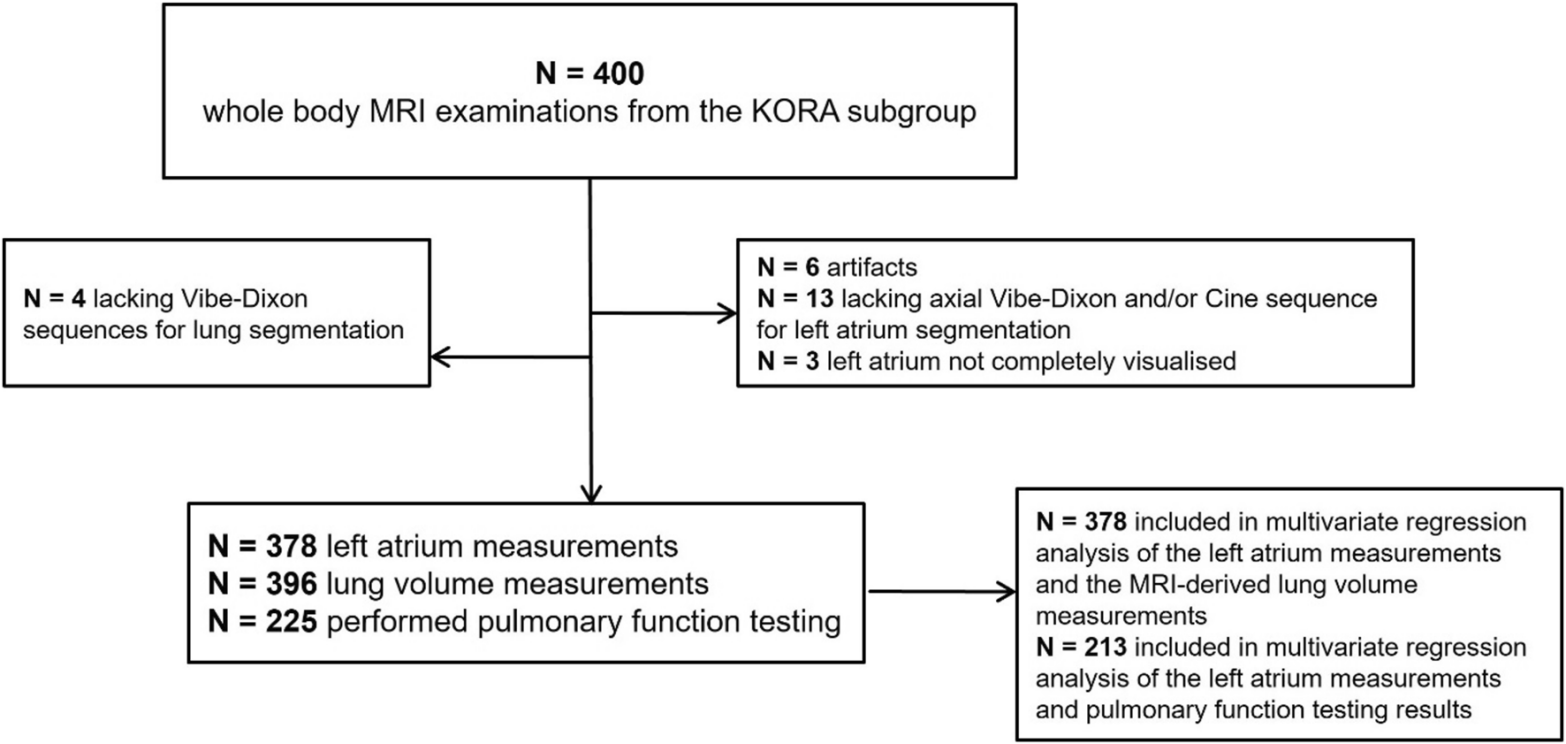
Flow chart depicting the study design. Of 400 participants undergoing whole-body MRI examinations, 378 participants were included in the left atrium measurements, 396 in the lung volume measurements, and 225 of the participants performed pulmonary function testing^12,14^. MRI: magnetic resonance imaging, KORA: Cooperative Health Research in the Region of Augsburg.

Our study was performed within the prospective cohort of the Cooperative Health Research in the Region of Augsburg (KORA)^15,16^. KORA is a population-based, longitudinal, epidemiological cohort study. It initially recruited 18,000 participants, divided into 4 subgroups (S1–S4) with follow-up health examinations^15^. The KORA-FF4 study (n = 2279) represents one of the follow-up examinations including participants of the S4-subgroup. A total of 400 participants from this KORA-FF4 cohort underwent whole-body MRI and PFT between June 2013 and September 2014^16,17^. Participants who agreed to undergo whole-body MRI examination were included in the KORA-MRI study. The exclusion criteria for this population-based study included the following: a history of cardiovascular disease (myocardial disease, stroke, revascularization therapy), age > 72 years, the presence of a non-MRI suitable implant, pregnancy, breastfeeding, claustrophobia, renal insufficiency, and known allergy to gadolinium compounds^16^. Of the 400 participants who underwent whole-body MR-imaging, 22 were excluded from the final analysis of the LA, either due to a lack of sequences (n = 13), artifacts (n = 6), or incomplete depiction of the LA in the acquired sequences (n = 3), leaving a total of 378 participants in which the LA was examined^14^. In four participants, the MR-imaging quality was inadequate, resulting in their exclusion from the automatic lung volume analysis, leaving 396 participants in which the lung volume was analyzed by MRI^12^. Of the 400 participants, 225 underwent PFT (Fig. 1).

The KORA-MRI study was approved by the Institutional Research Ethics Board of the Medical Faculty at Ludwig-Maximilian University Munich, and adhered to the principles outlined in the Helsinki Declaration on Human Research^18^.

### Clinical characteristics

The KORA FF4 examinations took place at the KORA study center and included interviews, health examinations, laboratory analysis, and medication records as described elsewhere^15,16^. Briefly, body surface area (BSA) was calculated using the Du Bois formula (BSA = 0.007184 * body height 0.725 * body weight 0.425), and smoking status was defined as never-smoker, ex-smoker, or current smoker; hypertension was defined as a systolic blood pressure of ≥ 140 mmHg or a diastolic blood pressure of ≥ 90 mmHg, as well as antihypertensive medication. Diabetes was defined according to the 1998 WHO criteria^19^.

### Pulmonary function tests

Pulmonary function assessments were conducted following the technical standards document developed by the American Thoracic Society and the European Respiratory Society^20^. A total of 225 participants underwent a minimum of two acceptable and reproducible PFT maneuvers using a pneumotachograph-type spirometer (MasterScope, Jaeger, Hoechberg, Germany)^12^.

### Whole-body magnetic resonance imaging

The participants underwent a whole-body MRI examination using a 3-Tesla MRI system (Magnetom Skyra, Siemens AG, Healthcare Sector, Erlangen, Germany). The detailed whole-body magnetic resonance imaging protocol has been extensively described in previous publications^16^. For the assessment of the maximum and minimum LA size, unenhanced ECG-synchronized CINE-steady-state free precession sequences in breath-hold technique were employed. The specific parameters included: slice thickness 8 mm, voxel size 1.5 × 1.5 mm^2^, field of view (FOV) 297 × 360 mm, matrix 240 × 160, repetition time (TR) 29.97 ms, echo time (TE) 1.46 ms, flip angle 62°^16^. For the analysis of the lung parameters and the LA area without gating, multiple breath-hold, 2-point DIXON T1-VIBE sequences were used, acquired in the submaximal inspiration breath-hold, and lasting 15 s^12,16^. For the LA area without cardiac gating, axial sequences were used with the following parameters: slice thickness 1.7 mm, voxel size 1.7 × 1.7 mm^2^, FOV 488 × 716 mm, matrix 256 × 256, TR 4.06 ms, TE 1.26 × 2.49 ms, 9° flip angle^16^. For the lung volume coronal sequences were used: slice thickness 3 mm, FOV 488 × 716, matrix 256 × 256, TR 4.06 ms, TE 1.26 ms^12^.

### MR-image analysis of the LA size

LA size was analyzed by a radiologist blinded for all other patients' data using the medical platform “NORA” (http://www.nora-imaging.com)^14^. Manual segmentation included delineation of the maximum and minimum LA area (LAmax, LAmin) on a single slice of gated 4-chamber view CINE-images, measured at end-systole and the end-diastole respectively. Quantification of the LA area in axial cross-section without ECG-gating was performed manually on a single axial slice at the level of the left ventricular outflow level and mitral valve in the opposed phase of VIBE-Dixon sequences. All the measurements of the LA excluded the pulmonary veins and included the LA appendage. As a substitute for the volume-based LA total ejection fraction, we established an area-based measurement termed the left atrium area fraction (LAaf). The LAaf was determined through the following equation: LAaf = (LAmax − LAmin)/LAmax. For the purpose of interreader- and intrareader variability testing, the same reader and a second, blinded, independent reader performed subsequent analysis after at least 2 months on 31 randomly chosen participants^14^.

### MR-image analysis of pulmonary volume

The analysis of pulmonary volume and automated processing of MRI data were conducted using a specified algorithm^12,21^. The lung segmentation algorithm included the correction of intensity inhomogeneities, initial extraction of a coarse region of interest delineating the airways, segmentation of both lungs and tracheal regions, extraction of the trachea with subsequent separation of the lungs into right and left lobes, and fine-tuning of pulmonary parenchyma. For the measurements, pulmonary vasculature extending beyond the margin contours of the mediastinum was included^12^. Two blinded readers independently validated the automatically achieved results for quality assurance^12^.

### Statistical analysis

The MRI-based cardiac and pulmonary data, pulmonary function measurements, and participants’ clinical characteristics are presented as arithmetic means with standard deviation (SD) for continuous variables or as counts and percentages for categorical variables with p-values from tests for trends across ordered groups. Scatter plots were used to display unadjusted correlations between LA parameters and MR-derived total lung volume and their distributions. Locally weighted regression lines were added to confirm linearity of the relations. Pearson correlation coefficients (r) were provided. Linear regression models were employed to evaluate the association between the LA measurements, treated as exposure variables and pulmonary function results as well as MRI-based lung volume measurements, treated as outcomes, providing β-coefficients with 95% confidence intervals (CI). First, the models were adjusted for BSA, while a second step included additional adjustments for age, sex, and smoking status. Finally, additional adjustments included diabetes status, HbA1c (glycated hemoglobin a1c), systolic and diastolic blood pressure, antihypertensive medication, total cholesterol, lipid-lowering medication, and glomerular filtration rate (GFR). A two-sided p-value < 0.05 was regarded as statistically significant. Statistical analyses were conducted using Stata 16.1 (Stata Corporation, College Station, TX, USA).

### Ethics approval and consent to participate

The KORA-MRI substudy was approved by the Institutional Research Ethics Board of the Medical Faculty of Ludwig-Maximilian University, Munich. The requirements of the Helsinki Declaration on human research were met. Informed written consent was obtained from each participant prior to the MRI exams.

## Results

Table 1 summarizes the clinical characteristics of the study population and the results of the cardiac and pulmonary MRI-analysis as well as the pulmonary function tests. The mean age of the population was 56.3 years (range 39 -73 years) and 42.3% (n = 160) were female. The subjects had an average body mass index (BMI) of 28.1 kg/ m^2^ (range 18.1–47.1 kg/ m^2^). Of the study population, 36.0% (n = 136) had never smoked, 20.9% (n = 79) were current smokers, and 163 participants (43.1%) had smoked before. The LA size derived from non-gated sequences measured 16.8 cm^2^ on average, and the maximal and minimal LA size derived from gated LA measurements were 19.6 cm^2^ and 11.9 cm^2^, respectively. The average total MRI-derived lung volume was 4.0 L, with an average of 2.2 L for the right lung and 1.8 L for the left lung. The average outcomes for PFT were 6.2 L for the total lung capacity, a residual lung volume of 2.1 L, and a forced vital capacity of 4.1 L. A FEV1/FVC ratio < 70% was reported for 48 of 213 participants.

**Table 1:** Characteristics of the study population.

	All n = 378	MRI-derived total lung volume			p-value
		Low (1.74–3.44L) n = 131	Medium (3.45–4.35L) n = 117	High (4.36–8.32L) n = 130	
BSA (m^2^)	1.95 (± 0.22)	1.86 (± 0.20)	1.95 (± 0.23)	2.04 (± 0.18)	< 0.001
BMI (kg/m^2^)	28.1 (± 4.9)	29.0 (± 5.2)	27.7 (± 5.0)	27.7 (± 4.5)	0.040
Age (years)	56.3 (± 9.2)	56.2 (± 8.9)	56.2 (± 9.4)	56.5 (± 9.5)	0.777
Sex (men)	218 (57.7%)	35 (26.7%)	66 (56.4%)	117 (90.0%)	< 0.001
Smoking status					
Never smoker	136 (36.0%)	61 (46.6%)	35 (29.9%)	40 (30.8%)	
Ex-smoker	163 (43.1%)	49 (37.4%)	60 (51.3%)	54 (41.5%)	
Current smoker	79 (20.9%)	21 (16.0%)	22 (18.8%)	36 (27.7%)	
Pack years	12.9 (± 18.0)	7.7 (± 12.5)	14.1 (± 18.3)	17.0 (± 21.1)	< 0.001
Hypertension	131 (34.7%)	45 (34.4%)	43 (36.8%)	43 (33.1%)	0.830
Systolic BP (mmHg)	120.8 (± 16.8)	117.2 (± 15.8)	121.3 (± 17.4)	123.9 (± 16.7)	0.002
Diastolic BP (mmHg)	75.3 (± 10.1)	74.4 (± 9.6)	75.3 (± 9.9)	76.1 (± 10.7)	0.180
Antihypertensive medications	98 (25.9%)	38 (29.0%)	30 (25.6%)	30 (23.1%)	0.275
Diabetes status					
No diabetes	228 (60.3%)	87 (66.4%)	74 (63.3%)	67 (51.5%)	
Prediabetes	99 (26.2%)	29 (22.1%)	28 (23.9%)	42 (32.3%)	
Diabetes	51 (13.5%)	15 (11.5%)	15 (12.8%)	21 (16.2%)	
HbA1c (%)	5.59 (± 0.75)	5.58 (± 0.65)	5.55 (± 0.62)	5.62 (± 0.93)	0.797
Alcohol use (g/day)	18.8 (± 24.1)	11.7 (± 17.8)	17.9 (± 19.6)	26.6 (± 30.3)	< 0.001
Total cholesterol (mg/dL)	218.5 (± 36.8)	221.4 (± 36.7)	215.1 (± 34.8)	218.6 (± 38.6)	0.442
HDL (mg/dL)	61.8 (± 17.7)	65.1 (± 17.5)	63.5 (± 19.0)	56.9 (± 15.7)	< 0.001
LDL (mg/dL)	140.1 (± 33.3)	140.5 (± 31.2)	135.7 (± 32.4)	143.7 (± 35.7)	0.558
Triglycerides (mg/dL)	133.6 (± 86.2)	128.1 (± 88.6)	127.1 (± 83.7)	144.8 (± 85.6)	0.054
Lipid-lowering medications	41 (10.9%)	18 (13.7%)	9 (7.7%)	14 (10.8%)	0.439
GFR (ml/min/1.73 m^2^)	86.7 (± 12.9)	86.9 (± 12.0)	86.6 (± 13.7)	86.6 (± 13.1)	0.683
Serum creatinine (mg/dL)	0.89 (± 0.16)	0.82 (± 0.14)	0.88 (± 0.17)	0.95 (± 0.14)	< 0.001
LA MRI measurements					
LAmax (cm^2^)	19.6 (± 4.5)	19.8 (± 4.3)	20.3 (± 4.9)	18.9 (± 4.3)	0.093
LAmin(cm^2^)	11.9 (± 3.5)	11.7 (± 3.5)	12.1 (± 3.8)	11.8 (± 3.1)	0.810
LA area fraction (%)	39.8 (± 9.2)	41.6 (± 9.0)	40.3 (± 9.3)	37.5 (± 8.7)	< 0.001
LA non-gated (cm^2^)	16.8 (± 4.0)	16.5 (± 3.8)	16.8 (± 4.3)	17.3 (± 3.8)	0.137
Lung MRI measurements	n = 378				
Total lung volume (L)	4.00 (± 1.11)	2.86 (± 0.40)	3.90 (± 0.25)	5.24 (± 0.71)	< 0.001
Right lung volume (L)	2.18 (± 0.58)	1.58 (± 0.23)	2.13 (± 0.14)	2.82 (± 0.38)	< 0.001
Left lung Volume (L)	1.82 (± 0.54)	1.28 (± 0.20)	1.76 (± 0.15)	2.42 (± 0.37)	< 0.001
Pulmonary function	n = 213				
FEV1 (L/s)	3.08 (± 0.78)	2.69 (± 0.69)	3.20 (± 0.70)	3.38 (± 0.77)	< 0.001
FVC (L)	4.13 (± 1.04)	3.46 (± 0.86)	4.32 (± 0.93)	4.68 (± 0.92)	< 0.001
FEV1/FVC (%)	74.9 (± 7.7)	77.9 (± 6.3)	74.2 (± 6.5)	72.3 (± 9.0)	< 0.001
Residual volume (L)	2.13 (± 0.40)	1.86 (± 0.36)	2.17 (± 0.34)	2.37 (± 0.32)	< 0.001
Total lung capacity (L)	6.22 (± 1.24)	5.32 (± 1.08)	6.44 (± 1.05)	6.93 (± 0.98)	< 0.001
Forced expiratory flow 25–75% (L/s)	2.46 (± 0.95)	2.43 (± 0.83)	2.45 (± 0.96)	2.52 (± 1.07)	0.683

The MRI-based lung volume was stratified in tertiles. Data are means and standard deviations for continuous variables and counts and percentages for categorical variables. P-values are from tests for trends across ordered groups. BMI: Body mass index, BSA: Body surface area, BP: Blood pressure, FEV1: Forced expiratory volume in 1 s, FVC: Forced vital capacity, GFR: Glomerular filtration rate, HbA1c: Glycated hemoglobin A1c, HDL: High density lipoprotein, LA: Left atrium, LA area fraction: Left atrium area fraction calculated as (LAmax-LAmin)/LAmax, LAmax: Maximum left atrium area, LAmin: Minimum left atrium area, LA non-gated: Left atrium area derived from axial, non-gated sequences, LDL: Low density lipoprotein.

### Association of left atrium size with MRI-based lung volume

**Fig. 2:**
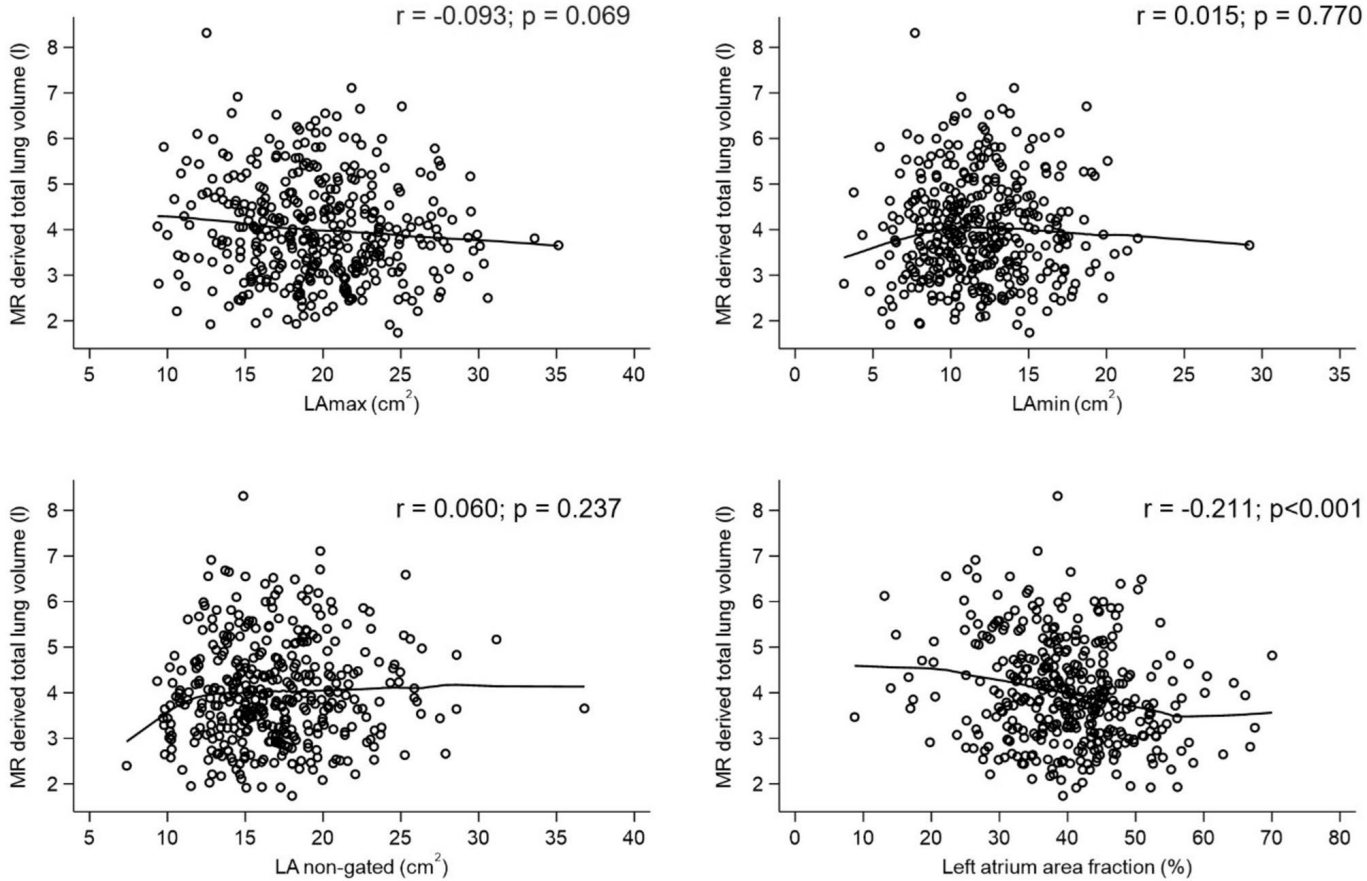
Scatter plot delineating the relationship between MRI-derived total lung volume in liters and the left atrium measurements. A locally weighted regression line indicates association between outcome and exposure. MRI: magnetic resonance imaging, LA: left atrium, LAmax: maximum left atrium area, LAmin: minimum left atrium area, LA area fraction: left atrium area fraction calculated as (LAmax-LAmin)/LAmax, LA non-gated: left atrium area derived from axial, non-gated sequences, r: Pearson correlation coefficients.

A total of 378 participants had both adequate LA measurements and MRI-derived lung volume measurements and were therefore included in the multivariate analysis (Fig. 1). Figure 2 shows the relationship between the unadjusted LA measurements and the MRI-derived lung volumes, with a Pearson correlation coefficient of r = − 0.211 (p < 0.001) for the MR-derived lung volume and left atrium area fraction, and r = − 0.093 (p = 0.069) for the MR-derived lung volume and LAmax. In the first model adjusted for BSA, a significant, inverse correlation between LAmax, and the LA area fraction with the MRI-based total lung volume was observed (β = − 0.04, p = 0.001, and β = − 0.02 p = 0.002, respectively). When further adjusting for age, sex, and smoking status, the significant association between LAmax, and LA area fraction persisted (β = − 0.03, p = 0–008 and β = − 0.01, p = 0.012, respectively), whilst the non-gated LA size was also significantly negatively associated with the MRI-derived total lung volume (β = − 0.03, p = 0.02). In the third model, additionally adjusted for diabetes status, HbA1c, systolic blood pressure, diastolic blood pressure, antihypertensive medication, total cholesterol, lipid-lowering medication, and GFR, the significant negative association of LAmax (β = − 0.03, p = 0.006), non-gated LA (β = − 0.03, p = 0.021) and LA area fraction (β = − 0.01, p = 0.012) with MRI-derived lung volumes remained. The minimal LA area, however, showed no significant association with the MRI-based lung volume. The results are presented in Table 2.

**Table 2:** Associations between left atrium measurements and MRI-derived lung measurements.

Total lung volume (l)						
	Model 1		Model 2		Model 3	
	β (95%CI)	p-value	β (95%CI)	p-value	β (95%CI)	p-value
LAmax (cm^2^)	− **0.04 (− 0.06; − 0.02**)	**0.001**	**− 0.03 (− 0.05; − 0.01)**	**0.008**	**− 0.03 (− 0.05; − 0.01)**	**0.006**
LAmin (cm^2^)	− 0.02 (− 0.05; 0.01)	0.201	− 0.02 (− 0.05; 0.01)	0.234	− 0.02 (− 0.05; 0.01)	0.198
LA non-gated (cm^2^)	− 0.02 (− 0.05; 0.01)	0.116	**− 0.03 (− 0.06; − 0.005)**	**0.020**	**− 0.03 (− 0.06; − 0.005)**	**0.021**
LA area fraction (%)	**− 0.02 (− 0.03; − 0.01)**	**0.002**	**− 0.01 (− 0.03; − 0.003)**	**0.012**	**− 0.01 (− 0.03; − 0.003)**	**0.012**

Model 1 Adjusted for BSA. Model 2: Model 1 and adjusted for age, sex, and smoking status. Model 3: Model 1, 2 and diabetes, HbA1c, systolic blood pressure, diastolic blood pressure antihypertensive medication, total cholesterol, lipid-lowering medication, GFR.

BSA: Body surface area, GFR: glomerular filtration rate, Hba1c: glycated hemoglobin a1c, LA: left atrium, LA area fraction: left atrium area fraction calculated as (LAmax-LAmin)/LAmax, LAmax: maximum left atrium area, LAmin: minimum left atrium area, LA non-gated: left atrium area derived from axial, non-gated sequences.

Significant values are in bold.

### Association of the left atrium with pulmonary function tests

Out of the total 400 participants, 213 underwent PFT and MRI-derived LA measurements, rendering them eligible for inclusion in the multivariate analysis (Fig. 1). No significant association between MRI-derived LA measurements and pulmonary function tests was found, even after full adjustment. The data are shown in Table 3.

**Table 3:** Associations between left atrium measurements and pulmonary function testing parameters.

	FEV1 (L/s)		FVC (L)		FEV1/FVC (%)		Residual volume (L)		Total lung capacity (L)		Forced expiratory flow 25–75%	
	β (95%CI)	p-value	β (95%CI)	p-value	β (95%CI)	p-value	β (95%CI)	p-value	β (95%CI)	p-value	β (95%CI)	p-value
Model 1												
LAmax (cm^2^)	− 0.007 (− 0.028; 0.013)	0.474	− 0.015 (− 0.042; 0.012)	0.269	0.069 (− 0.168; 0.306)	0.568	0.001 (− 0.01; 0.013)	0.839	− 0.01 (− 0.042; 0.022)	0.543	0.001 (− 0.027; 0.029)	0.938
LAmin (cm^2^)	− 0.015 (− 0.044; 0.013)	0.278	− 0.024 (− 0.062; 0.013)	0.199	0.082 (− 0.247; 0.411)	0.623	− 0.004 (− 0.02; 0.012)	0.654	− 0.014 (− 0.059; 0.030)	0.527	− 0.006 (− 0.045; 0.033)	0.751
LA non-gated (cm^2^)	− 0.004 (− 0.029; 0.021)	0.761	− 0.013 (− 0.046; 0.02)	0.447	0.159 (− 0.129; 0.447)	0.277	0.001 (− 0.012; 0.015)	0.841	0.003 (− 0.036; 0.041)	0.895	0.001 (− 0.033; 0.035)	0.967
LA area fraction (%)	0.004 (− 0.006; 0.015)	0.447	0.003 (− 0.011; 0.017)	0.624	− 0.001 (− 0.124; 0.122)	0.989	0.003 (− 0.003; 0.009)	0.260	0.000 (− 0.016; 0.017)	0.98	0.005 (− 0.01; 0.019)	0.523
Model 2												
LAmax (cm^2^)	0.007 (− 0.009; 0.023)	0.375	0.007 (− 0.012; 0.027)	0.454	0.003 (− 0.227; 0.232)	0.981	0.005 (− 0.005; 0.016)	0.295	0.011 (− 0.012; 0.034)	0.357	0.007 (− 0.02; 0.034)	0.627
LAmin (cm^2^)	0.005 (− 0.018; 0.027)	0.686	0.006 (− 0.021; 0.033)	0.665	0.006 (− 0.313; 0.324)	0.971	0.001 (− 0.013; 0.015)	0.889	0.013 (− 0.02; 0.046)	0.438	0.003 (− 0.035; 0.04)	0.889
LA non-gated (cm^2^)	0.007 (− 0.013; 0.027)	0.481	0.002 (− 0.022; 0.026)	0.853	0.146 (− 0.132; 0.424)	0.302	0.002 (− 0.01; 0.014)	0.738	0.014 (− 0.014; 0.043)	0.328	0.008 (− 0.025; 0.041)	0.616
LA area fraction (%)	0.003 (− 0.005; 0.011)	0.446	0.003 (− 0.008; 0.013)	0.623	− 0.005 (− 0.122; 0.113)	0.938	0.004 (− 0.001; 0.009)	0.147	0.000 (− 0.012; 0.012)	0.996	0.004 (− 0.01; 0.018)	0.608
Model 3												
LAmax (cm^2^)	0.005 (− 0.011; 0.021)	0.530	0.004 (− 0.015; 0.024)	0.649	− 0.003 (− 0.237; 0.231)	0.979	0.003 (− 0.007; 0.013)	0.538	0.004 (− 0.019; 0.026)	0.758	0.008 (− 0.02; 0.035)	0.590
LAmin (cm^2^)	0.005 (− 0.017; 0.027)	0.675	0.006 (− 0.021; 0.033)	0.659	0.002 (− 0.322; 0.326)	0.992	0.001 (− 0.013; 0.015)	0.930	0.010 (− 0.022; 0.041)	0.550	0.005 (− 0.033; 0.043)	0.804
LA non− gated (cm^2^)	0.006 (− 0.014; 0.026)	0.554	0.001 (− 0.023; 0.025)	0.915	0.124 (− 0.163; 0.411)	0.396	0.001 (− 0.011; 0.014)	0.818	0.010 (− 0.018; 0.038)	0.483	0.01 (− 0.024; 0.044)	0.570
LA area fraction (%)	0.002 (− 0.006; 0.01)	0.687	0 (− 0.01; 0.01)	0.992	0.003 (− 0.117; 0.123)	0.964	0.002 (− 0.003; 0.007)	0.474	− 0.004 (− 0.016; 0.007)	0.485	0.004 (− 0.01; 0.018)	0.595

Model 1 Adjusted for BSA. Model 2: Model 1 and adjusted for age, sex, and smoking status. Model 3: Model 1, 2 and diabetes, HbA1c, systolic blood pressure, diastolic blood pressure antihypertensive medication, total cholesterol, lipid-lowering medication, GFR.

BSA: Body surface area, FEV1: forced expiratory volume in 1 s, FVC: forced vital capacity, GFR: glomerular filtration rate, Hba1c: glycated hemoglobin a1c, LA: left atrium, LA area fraction: left atrium area fraction calculated as (LAmax-LAmin)/LAmax, LAmax: maximum left atrium area, LAmin: minimum left atrium area, LA non-gated: left atrium area derived from axial, non-gated sequences.

## Discussion

In this population-based study of participants without overt cardiovascular disease, increased LA size and function were associated with decreased MRI-based lung volumes. The maximum LA area in sequences with ECG-gating, the LA area in measurements without ECG-gating, and the LA area-derived function were all inversely associated with the MRI-derived total lung volume. However, there was no association observed with PFT parameters. In this study, we demonstrated that within a healthy study population, there seem to be common cardiopulmonary changes that can be simultaneously assessed by whole-body MRI. Subclinical cardiopulmonary changes might be detected by whole-body MRI prior to clinical symptoms and changes on PFT.

Our study showed an inverse association of LAmax, non-gated LA, and the LA area fraction with the MRI-derived total lung volume. A previous study in the same study subgroup showed an inverse association of left ventricular end-diastolic volume and left and right ventricular stroke volumes with MRI-derived lung volumes (β = − 0.14, β = − 0.14, β = − 0.11, all p = 0.01)^13^. This may point to common subclinical structural and functional alterations of the LA, ventricles, and lung volumes measured by whole-body MRI. Numerous cardiovascular diseases such as HFpEF and various lung diseases share established risk factors (such as age, obesity, smoking), and cardiac and pulmonary diseases often coexist; however, it has also been proposed that heart and lung function interact^5^. Although there is not an established direct relationship between impaired LA function and lung volumes, elevated LV filling pressures often manifest with enlarged LA volume due to elevated LA filling pressures^1–3^. Increased LA filling pressure is reflected in the pulmonary vascular bed with attendant pulmonary symptoms—where MRI-derived lung volume and pulmonary function tests like spirometry could assist in differentiating underlying cardiac versus pulmonary pathology^3–6^. Here, pulmonary function tests could perhaps be more sensitive than MRI-derived lung volume in evaluating overt obstructive lung disease^6^, however, our results show the additional incremental value of MRI-based lung volume in patients free of cardiovascular disease. Previous studies explored the cardiopulmonary axis in patients suffering from various symptomatic diseases. In patients with HFrEF, a clear relationship between the severity of the heart failure and a decrease in lung size was reported, possibly due to the resulting cardiomegaly^22^. Conversely, it was reported that in patients with chronic obstructive pulmonary disease (COPD), all cardiac chambers decreased with increasing GOLD stage, possibly due to lung hyperinflation^23^. Despite the absence of overt cardiovascular disease within our study group, the morphological changes observed were similar to those typically found in patients with manifest disease. This may be attributed in part to the shared thoracic cavity housing both lungs and heart. However, the absence of overt disease or lung function changes provides an opportunity to detect these changes in a subclinical state.

Our results in cardiovascular healthy individuals revealed no significant association between changes in the LA size or function with PFT parameters. Previous studies have investigated lung function changes in patients with manifest HFpEF. Andrea et al. reported lung function alterations in 94% (n = 69) of patients with newly diagnosed HFpEF with and without previous lung disease^5^. These findings were confirmed in patients within a larger study population (n = 1194) with and without COPD^6^. In earlier studies, lung function alterations in patients with HFpEF were a predictive marker for mortality independent of COPD^6,24^, highlighting the utility of PFT in this patient group. Population-based MRI studies have the potential to enhance our comprehension of HFpEF development and progression and to detect early subclinical stages. This approach may facilitate earlier risk modification and treatment interventions.

Traditionally, pulmonary imaging relies heavily on computed tomography (CT) due to its speed, availability, and detailed visualization of the lung parenchyma^25^. However, the utilization of whole-body MRI studies, with precise pulmonary and cardiac examination modalities, is on the rise, allowing radiation-free cross-sectional studies in individuals without cardiopulmonary disease^8–11^. An important aspect to consider is the different acquisition techniques of PFT and MRI-derived lung function techniques; while PFT is performed under maximal effort, in an upright sitting position, MRI is performed in a quiet resting, supine position; these positional differences may influence the results^12,13^. Within the same study cohort, it was previously shown that the MRI-derived lung function volume reflected 124.4 ± 27.9% of the functional residual capacity as calculated from reference equations^12^, however, variations in dynamic lung volume measurements like tidal volume and functional residual capacity which could be influenced by volume interdependencies within the thoracic cavity were not evaluated in our study. Furthermore, while PFT follows a strict predefined breathing regimen, MRI-derived lung volumes are acquired with loose standard breathing instructions^12^. Previous studies have shown that lung volumes detected from cross-sectional imaging represent submaximal inspiration and not a total lung capacity that is achieved by maximal inspiration^26^.

However, lung volume measurements derived from whole-body-MR imaging are independently associated with residual volume and FEV1/FVC (β = 0.50, p = 0.04 and β = − 0.02, p = 0.02) indicating its diagnostic utility^12^.

Cardiac magnetic resonance imaging (cMRI) employs ECG-gating to acquire dynamic images, mitigating artifacts arising from cardiac motion. These specialized protocols require general availability and expertise.

Previous studies have demonstrated that the LA can be effectively quantified using routinely acquired axial MR images of the thorax without the need for ECG gating^14^. Additionally, it was observed that similar cardiovascular risk factors were associated not only with LA measurements obtained through ECG-gated imaging but also with those derived from non-gated LA assessments^14^. Our investigations have substantiated these findings, revealing consistent associations of LA measurements irrespective of the utilization of gating.

The strengths of this study include its use of advanced 3-Tesla MR-imaging using standardized protocols, imaging processing, and detailed health parameters from health examinations, laboratory analysis, and medication. Our study group was free of overt cardiovascular disease enabling the detection of subclinical structural and functional changes.

Limitations of the study include the lack of LA volume analysis due to using a standardized protocol. Volumetric assessment of the LA is not routinely performed, and an adequate protocol would prolong the cMRI by ~ 6 min^27^. Furthermore, due to the cross-sectional analysis design, no follow-up information is available to determine HFpEF disease development or progression. In addition, the study population included a rather small population group. While our study provides valuable insights, expanding these findings through additional research in larger cohort studies will be beneficial for a more comprehensive understanding.

## Conclusion

In this population-based whole-body MRI study, we observed an association between subclinical LA impairment and MRI-based lung volume but not between subclinical LA impairment and PFT parameters. The simultaneous evaluation of LA size, function, and lung volume using MRI could offer valuable insights, particularly in patients with a high probability of subclinical HFpEF, where cardiopulmonary symptoms are a significant concern. This approach may enhance our understanding of the interplay between heart and lung pathologies.

## Data Availability

Restrictions apply to the availability of the data generated or analyzed during this study to preserve patient confidentiality or because they were used under license. The corresponding author will, on request, detail the restrictions and any conditions under which access to some data may be provided.

## Abbreviations

BMI: Body mass index
BSA: Body surface area
BP: Blood pressure
CI: Confidence interval
CT: Computed tomography
cMRI: Cardiovascular magnetic resonance imaging
COPD: Chronic obstructive pulmonary disease
ECG: Electrocardiography
FEV1: Forced expiratory volume in 1 s
FOV: Field of view
FVC: Forced vital capacity
GRF: Glomerular filtration rate
HbA1c: Glycated hemoglobin a1c
HFpEF: Heart failure with preserved ejection fraction
HFrEF: Heart failure with reduced ejection fraction
KORA: Cooperative Health Research in the Region of Augsburg
LA: Left atrium
LA non-gated: Left atrium area derived from axial, non-gated sequences
LAaf: Left atrium area fraction
LAmax: Maximum left atrium area
LAmin: Minimum left atrium area
LV: Left ventricle
MRI: Magnetic resonance imaging
PFT: Pulmonary function testing
RV: Right ventricle
SD: Standard deviation
TE: Echo time
TR: Repetition time
